# Supplementary material for: Precise Printing of Microfiber Scaffold with Gelatin Methacryloyl (GelMA)/Polyethylene Oxide (PEO) Bioink
Source: Bioengineering (Basel). 2023 Jan 18;10(2):130. doi: 10.3390/bioengineering10020130 (PMC9952242; doi:10.3390/bioengineering10020130)
Supplement: Supplementary file 1 [file bioengineering-10-00130-s001.zip › bioengineering-2150464-supplementary.pdf]

Supplementary Information

Precise Printing of Microfiber Scaffold with GelMA/PEO Bioink

Supplementary Note 1 | Microfiber scaffold separation from the glass sheet

The electrospun microfiber scaffold was photocrosslinked in the ammonium sulfate semi-saturated solution system. The blue light irradiation duration was set as 1 minute. After the crosslinking was completed, the glass sheet was transferred into clean deionized water. The printed microfiber scaffold would automatically separate from the glass sheet. The glass sheet could be then removed with tweezers and the scaffold was stored in clean deionized water for later use.

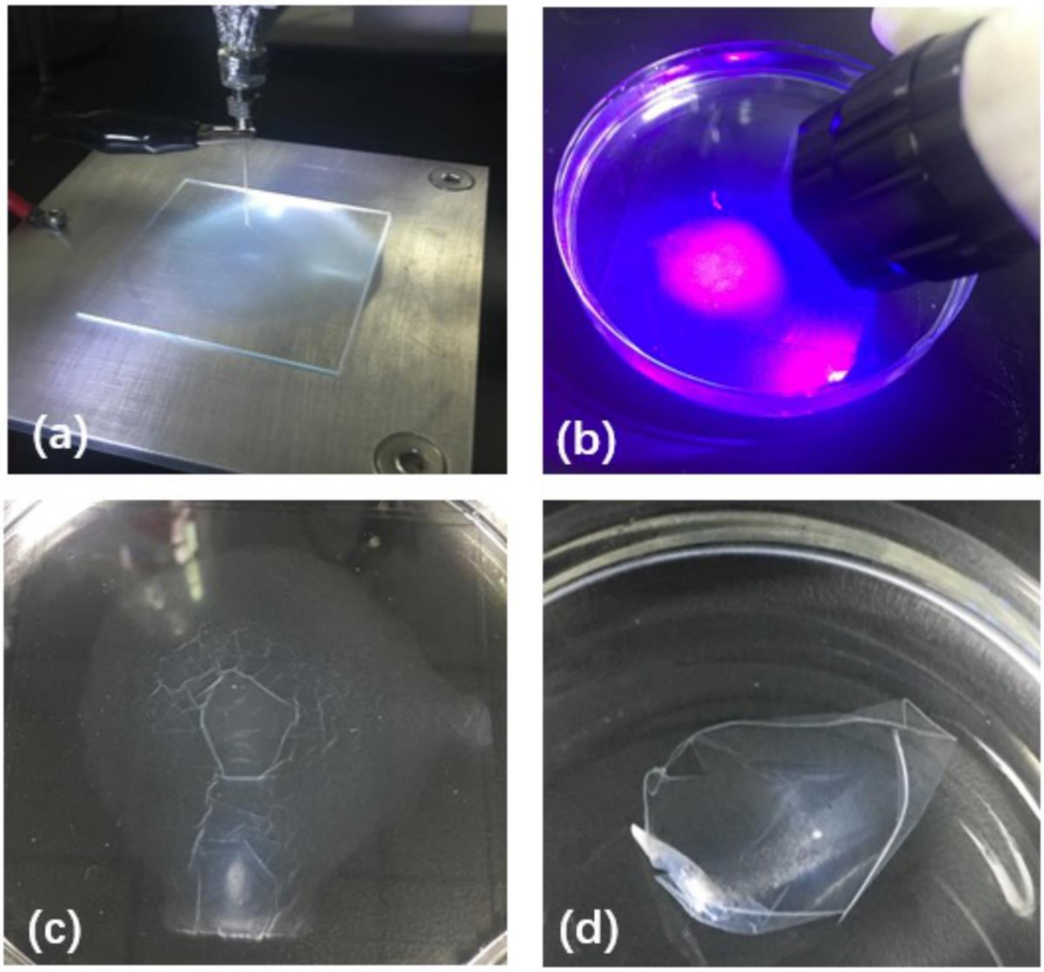

Figure S1 Microfiber scaffold separation from the glass sheet. a) Electrospinning. b) Photocrosslinking. c) Scaffold separated from glass sheet. d) Scaffold restored in the clean deionized water.

Supplementary Note 2 | Degradation of crosslinked GelMA/PEO hydrogel

The degradability of hydrogels plays an important role in the application of biological tissue scaffolds, and good tissue scaffolds can be naturally degraded after supporting cell growth, and finally form new tissues constructed only from autologous cells. The results of in vitro degradation experiments showed that each group of GelMA/PEO hydrogels could be fully degraded by type II

collagenase after 30 hours. However, there were slight differences in degradation speed due to different PEO content. In the initial stage, the group with higher PEO concentration degraded more quickly than the pure GelMA group. In the first 5 hours, hydrogels with a 2% (w/v) PEO displayed the slowest degradation speed, and then the degradation accelerated and its remaining mass after 15 h was the lowest in the 5 groups. The experimental results demonstrated that we could control the degradation speed by adjusting the PEO content.

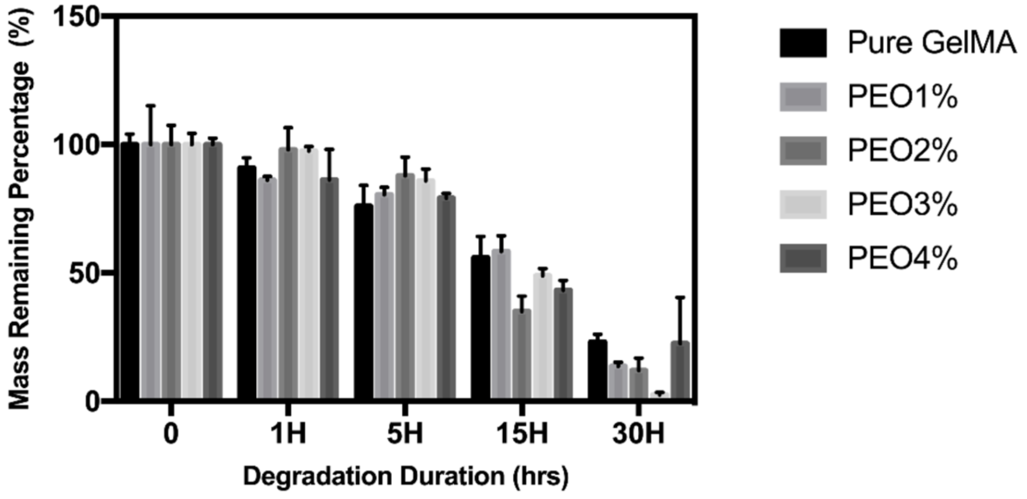

Figure S2 Degradation of crosslinked GelMA/PEO hydrogel.

Supplementary Note 3 | Capillary network printing with NEF direct writing

Red fluorescent microspheres with a diameter of 0.1 μm was added into the GelMA/PEO bioink to print a capillary network. The network G-code was written by researcher rather than the slicing software. Because the capillary

network model was more complex and could not be printed in one stroke, the strategy of repeatedly printing some parts of the routine was adapted to solve this problem. After printing, the sample was placed under a confocal microscope for observation, and the results showed that the capillary network could be successfully printed with the NEF direct writing method and the GelMA/PEO bioink, indicating their feasibility to prepare biomimetic microstructures.

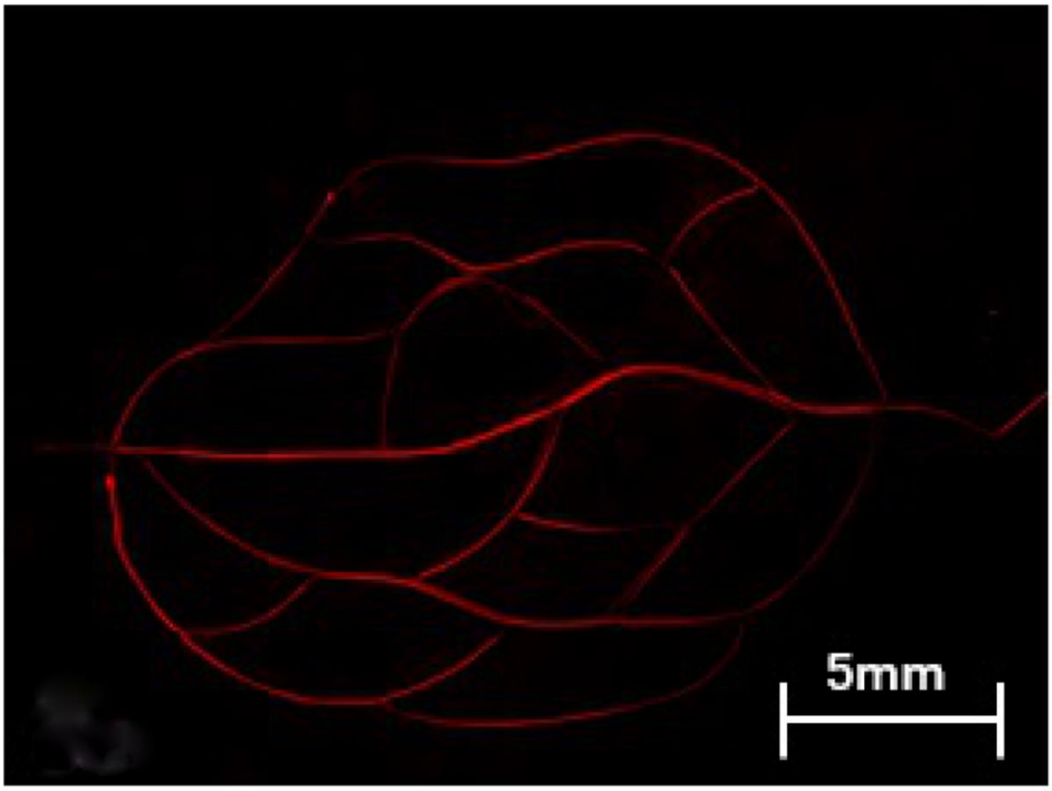

Figure S3 Capillary network printing with NEF direct writing

Supplementary Note 4 | Capillary network printing with NEF direct writing

Due to the close printing distance and incomplete volatilization of the bioink,

the diameter of the microfiber prepared by NEF direct writing was significantly larger than that of FEF electrospinning, which could reach 40 μm and is close to the cell scale. Here, green fluorescent microspheres with a diameter of 0.1 μm was added into the GelMA/PEO bioink to explore the ability to generate single cell microfibers. The experimental results showed that only one particle appeared at most in each section of the fiber (Figure S4 (h)), which could better simulate the printing effect of single cells and had good application prospects in the fields of single-cell cloning and cell behavior observation. Moreover, small scale grids, polygons, spirals and other shapes were constructed to simulate single-cell printing, and the experimental results showed that each green fluorescent microspheres represented a cell, and the green fluorescent microspheres distribution was relatively uniform in each pattern, basically each particle appearing in only one section.

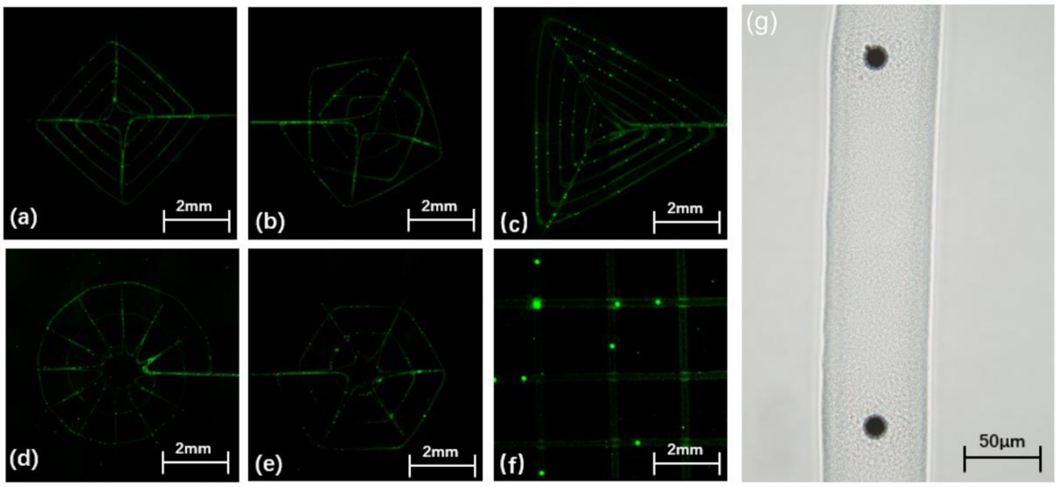

Figure S4 Single-cell microfiber printing with NEF direct writing. (a)-(f) Single-cell microfibers with different complex morphology. (g) The single microsphere image in high magnification.

Supplementary Note 5 | Cell viability on FEF electrospun microfiber scaffold.

Cell viability on the FEF electrospun microfiber scaffold was performed using a Live/Dead cell staining kit. The calcein-AM in the reagent could label the live cells and propidium iodide in the reagent could label dead cells. Then images were captured with a confocal fluorescence microscope and introduced to analysis software ImageJ. The cell viability was calculated by comparing the number of the green and red dots. The formula is as follows:

$$\frac{N_g}{N_r + N_g} \times 100\%$$

where N_g represented the number of surviving cells (green dots) in the field of view and N_r represented the number of dead cells (red dots).

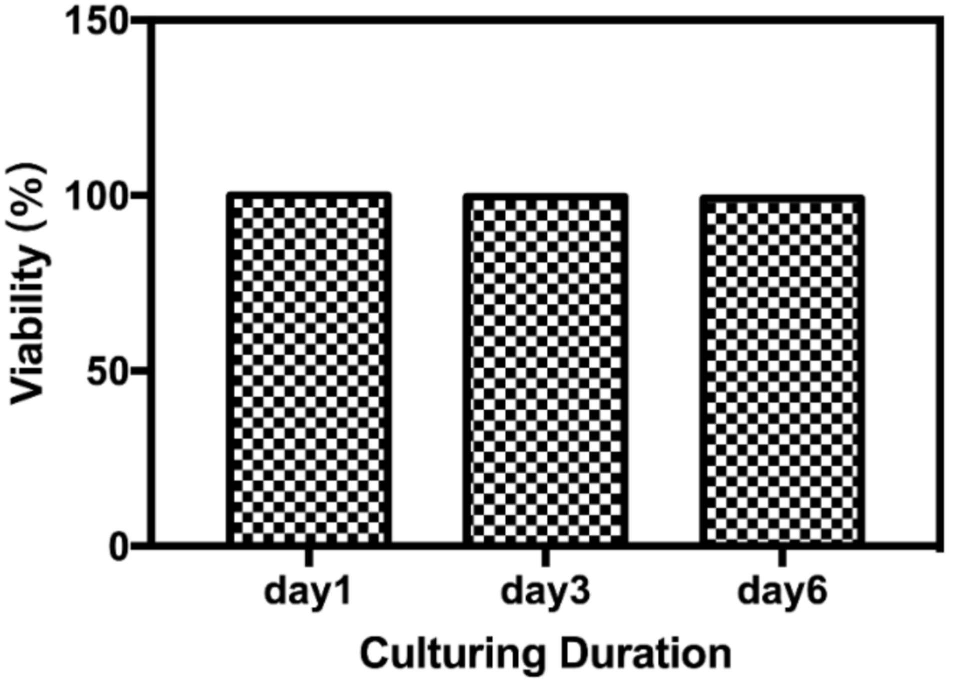

Figure S5 HUVECs viability result on the FEF microfiber scaffold.
